# Theoretical Analysis of Constant Voltage Mode Membrane Capacitive Deionization for Water Softening

**DOI:** 10.3390/membranes11040231

**Published:** 2021-03-24

**Authors:** Xin Zhang, Danny Reible

**Affiliations:** 1 Department of Chemical Engineering, Texas Tech University, Lubbock, TX 79409-3121, USA; xin1992.zhang@ttu.edu; 2 Department of Civil, Environmental and Construction Engineering, Texas Tech University, Lubbock, TX 79409-1023, USA

**Keywords:** water softening, membrane capacitive deionization (MCDI), selectivity, water recovery, specific energy consumption (SEC)

## Abstract

Water softening is desirable to reduce scaling in water infrastructure and to meet industrial water quality needs and consumer preferences. Membrane capacitive deionization (MCDI) can preferentially adsorb divalent ions including calcium and magnesium and thus may be an attractive water softening technology. In this work, a process model incorporating ion exclusion effects was applied to investigate water softening performance including ion selectivity, ion removal efficiency and energy consumption in a constant voltage (CV) mode MCDI. Trade-offs between the simulated Ca^2+^ selectivity and Ca^2+^ removal efficiency under varying applied voltage and varying initial concentration ratio of Na^+^ to Ca^2+^ were observed. A cut-off CV mode, which was operated to maximize Ca^2+^ removal efficiency per cycle, was found to lead to a specific energy consumption (SEC) of 0.061 kWh/mole removed Ca^2+^ for partially softening industrial water and 0.077 kWh/m^3^ removed Ca^2+^ for slightly softening tap water at a water recovery of 0.5. This is an order of magnitude less than reported values for other softening techniques. MCDI should be explored more fully as an energy efficient means of water softening.

## 1. Introduction

The majority of natural waters contain a certain amount of hardness (i.e., divalent ions, primarily calcium and magnesium), causing potential fouling, scaling and taste issues. Excess intake of calcium and magnesium inhibits the adsorption of other essential elements and may cause diarrhea, while inadequate uptake of either calcium or magnesium also poses health threats [1]. Removal of hardness is common in waters for industrial, agricultural and domestic use.

Traditional techniques used for softening hard waters include ion-exchange [2], chemical and electrochemical precipitation [3,4], nanofiltration [5,6] and electro-membrane processes [7]. Capacitive deionization (CDI) removes charged particles in solution by attracting them toward the oppositely charged porous electrodes and temporarily holding them in the electric double layer (EDL) formed near the electrodes’ surface [8,9]. CDI is applicable to water softening due to the preferential electrosorption of divalent hardness ions over monovalent ions [10,11] and the technology has been investigated for softening brackish waters [12,13,14,15,16,17,18].

Membrane CDI (MCDI) inserts ion-exchange membrane (IEM) between the electrodes and porous spacer to enhance desalination performance and improve energy efficiency [19]. Specifically, a cation-exchange membrane (CEM) is assembled onto the cathode and an anion-exchange membrane (AEM) is assembled onto the anode. IEM facilitates counter-ions’ transport but inhibits co-ions’ penetration. IEM improves ion transport rate and increases the flux of hardness ions over that of sodium ions, enabling a faster and more efficient water softening in MCDI compared to conventional CDI [20]. Fouling and scaling issues are largely alleviated in MCDI compared to conventional CDI and other desalination techniques due to the protection of the IEM and the reversed ion transport direction during regeneration [21,22].

MCDI water softening performance is determined by feed water chemistry, hydrated ion radius, ion valence, electrode and IEM construction and materials, operating modes and operating conditions [15,16,23,24]. Hou and Huang [15] observed preferential adsorption of ions with smaller hydrated ion radius, larger charge valence and higher initial concentration in a batch mode CDI. IEM selective permeation toward specific ions is governed by the affinity of IEM toward the ions, ion concentration outside the IEM and ion mobility inside the IEM and can be enhanced by membrane surface modification [25]. Specific IEM modifications such as replacing CEM with Ca-alginate [16] and coating polyelectrolyte multilayers onto the CEM [24] have been employed to tune ion selectivity in MCDI. He et al. [23] observed that selectivity of calcium ions was enhanced under lower applied current and shorter hydraulic retention time in a flow-electrode CDI.

Substitution of monovalent ions (e.g., sodium) by divalent ions has been observed near adsorption saturation of the electrode in constant voltage (CV) mode CDI [13,14,26]. Zhao et al. [14] explained this selectivity using the Boltzmann distribution to indicate that close to saturation, the accumulated potential in EDL becomes significant and ion selectivity is dominated by valence. Ion selectivity in a constant current (CC) mode, however, depends more on the ion transport through cell elements since electrode saturation is not reached in CC mode. Wang and Lin [20] observed a correlation between ion selectivity and ion flux through the IEM in CC mode MCDI resulting from differences in partition coefficient, effective diffusion coefficient and ion concentration.

A number of process models have been developed for depicting dynamic ion transport and adsorption in (M)CDI but all to-date have been limited to treating ions as point charges [20,27,28,29,30,31,32]. Yet treating ions as point charges is unable to capture excluded ion volume effects [33], limiting the ability to simulate ion adsorption and selectivity in multicomponent solutions containing ions with different hydration radii. Suss [34] introduced an excess chemical potential term into the modified Donnan theory to correct ion concentration in macropores and micropores for the available volume (pore volume minus the excluded volume of each ion that are inaccessible to other ions). Guyes et al. [35] employed Suss’s theory and incorporated the effects of the attached surface charges on the electrode, successfully capturing the experimentally observed preferential adsorption toward the smaller ions in a batch mode CDI using functionalized electrode.

Trade-offs between calcium selectivity and calcium removal efficiency were discovered in CC mode MCDI [20]. However, hardness removal efficiency is a more important performance metric in water softening. CV mode MCDI was observed to reach high salt removal efficiency by reversing electrode polarity at maximum salt removal efficiency (termed here as “cut-off” mode) [32]. In this work, water softening performance of a CV mode MCDI is theoretically explored by investigating the selectivity and removal efficiency of hardness ions and the energy behaviors. The objectives of this work are to 1) extend our previously built MCDI process model [32] to incorporate excluded ion volume effects and compare the respective simulation results to those achieved with the original model, 2) compare selectivity and removal efficiency of hardness ion under varying operating duration, 3) explore the trade-offs between selectivity and removal efficiency of hardness ions in a cut-off CV mode MCDI and 4) analyze the cell performance, energy consumption and feasibility of applying MCDI to soften waters of various purposes, including industrial cooling tower blowdown water and domestic tap water.

## 2. Model Framework

Our model is based on a single-pass CV mode MCDI with flow direction in parallel with the electrodes [32]. Common components in brackish waters, including the divalent ions typically responsible for hardness, calcium and magnesium, their reported hydrated radii [36] and diffusion coefficients [37] are displayed in Table 1.

The basic modeling approach is described in [32]. Here, we focus on the modifications necessary to include excluded ion volume effects. We employ a modification to Donnan theory considering an excess chemical potential difference term [34]:

(1) $$c_{mi,i}=c_{ma,i}exp\left(-\frac{z_{i}F\varphi_{d}}{RT}-\Delta \mu_{i}^{ex}\right)$$

where *c_mi,i_* is the concentration of species *i* in micropores, *c_ma,i_* is the concentration of species *i* in macropores, *z_i_* is the ion valence of species *i*, *F* is the Faraday’s constant (96,485 C/mol), *φ_d_* is the Donnan potential of micropores, *R* is the universal gas constant (8.314 J/mol/K), *T* is the ambient temperature and $\Delta \mu_{i}^{ex}$ is the difference of the excess chemical potentials in micropores and the adjacent macropores:

(2) $$\Delta \mu_{i}^{ex}=\mu_{mi,i}^{ex}-\mu_{ma,i}^{ex}$$

where $\mu_{mi,i}^{ex}\;$ and $\mu_{ma,i}^{ex}$ are the excess chemical potentials in micropores and the adjacent macropores, respectively, accounting for ion exclusion effects.

For a multi-component system containing hard-sphere ions with different hydrated ion radii, the excess chemical potential can be analytically expressed by Boublik-Mansoori-Carnahan-Starling-Leland (BMCSL) equation [34]:

(3) $$\mu_{j,i}^{ex}=-\left(1+\frac{2\xi_{2}^{3}d_{i}^{3}}{\phi^{3}}-\frac{3\xi_{2}^{2}d_{i}^{2}}{\phi^{2}}\right)\ln\left(1-\phi \right)+\frac{3\xi_{2}d_{i}+3\xi_{1}d_{i}^{2}+\xi_{0}d_{i}^{3}}{1-\phi }+\frac{3\xi_{2}d_{i}^{2}}{{\left(1-\phi \right)}^{2}}\left(\frac{\xi_{2}}{\phi }+\xi_{1}d_{i}\right)-\xi_{2}^{3}d_{i}^{3}\frac{\phi^{2}-5\phi +2}{\phi^{2}{\left(1-\phi \right)}^{3}}$$

where *j* represents *mi* and *ma* and *d_i_* is the hard-sphere diameter of species *i*, which is related to the reported hydrated ion diameter *d_h,i_* through a constant factor *C* to fit the experimental data.

(4) $$d_{i}=Cd_{h,i}$$

*ϕ* is the volume fraction of all the ions:

(5) $$\phi =\underset{i}{\sum }\phi_{i}=\underset{i}{\sum }\frac{\pi d_{i}^{3}}{6}c_{j,i}N_{a}$$

where *c_j,i_* is the concentration of species *i* in location *j*, macropores or micropores and *N_a_* is the Avogadro’s constant (6.022 × 10^23^ mol^−1^).

*ξ_k_* is expressed by:

(6) $$\xi_{k}=\underset{i}{\sum }\phi_{i}d_{i}^{k-3}$$

External resistance effects are included in the model with the relation:

(7) $$V_{cell}=V_{e}+I_{ext}R_{ext}$$

where *V_cell_* is the applied voltage, *V_e_* is the electric potential drop on the electrode pair, *R_ext_* is the external resistance and *I_ext_* is the external current [27].

In this study, MCDI is assumed symmetric with identical cathode and anode and identical CEM and AEM. Detailed MCDI device parameters and operating conditions based on a single cell unit are listed in Table 2. The parameters marked with * and # are only used in the example of industrial cooling tower blowdown water softening (Section 3.3.1) and domestic tap water softening (Section 3.3.2).

## 3. Results and Discussion

### 3.1. Excluded Ion Volume Effects

**Figure 1:**
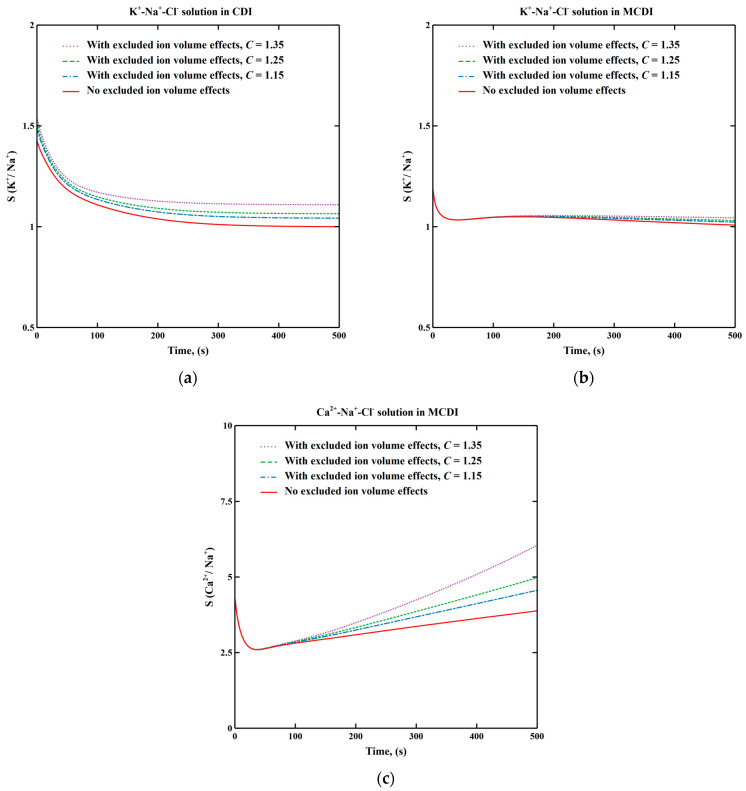
Transient K^+^ selectivity in K^+^-Na^+^-Cl^−^ solution with and without excluded ion volume effects during desalination in (**a**) capacitive deionization (CDI) and (**b**) membrane capacitive deionization (MCDI). (**c**) Transient Ca^2+^ selectivity in Ca^2+^-Na^+^-Cl^−^ solution with and without considering excluded ion volume effects during desalination in MCDI. Adjustable variable *C* is varied from 1.15 to 1.35. Applied voltage is 0.3 V. Feed concentration of each cation is 20 mol/m^3^.

In a multicomponent saline solution, selectivity of cationic species *i* is usually defined as the ratio of the removal efficiency of the cationic species *i* to that of sodium ions [20]:

(8) SiNa+=Δcic0,iΔcNa+c0, Na+=ηiηNa+. 

where *S*(*i/Na^+^*) is the selectivity of the cationic species *i*, *c_0,i_* is the initial concentration of the cationic species *i*, *c_0,Na_^+^* is the initial concentration of sodium ions, Δ*c_i_* is the concentration reduction of the cationic species *i* during desalination, Δ*c_Na_^+^* is the concentration reduction of sodium ions during desalination, *η_i_* is the removal efficiency of the cationic species *i* and *η_Na_^+^* is the removal efficiency of sodium ions. Ion selectivity in (M)CDI is calculated based on the simulated effluent concentration with and without considering excluded ion volume effects by setting an inlet concentration of 20 mol/m^3^ for all cations. The respective transient selectivity curves of K^+^ and Ca^2+^ are displayed in Figure 1.

In order to verify this extended process model, transient K^+^ selectivity in K^+^-Na^+^-Cl^−^ solution in CDI considering excluded ion volume effects is compared with that of ignoring excluded ion volume effects in Figure 1a. The use of these monovalent ions in this analysis allows us to explore only the effects of ionic radii separate from valence.

As shown in Figure 1a, excluded ion volume effects slightly increases K^+^ selectivity. At the beginning of charging, K^+^ selectivity is higher than 1, which is attributed to the higher diffusivity of K^+^ compared to Na^+^. When electrode saturation is reached (times greater than 300 s in this simulation), macropore concentration becomes uniform and identical to feed water concentration and selectivity in the absence of excluded ion volume effects approaches unity. With excluded ion volume effects, the smaller hydrated ion size of K^+^ increases adsorption of K^+^ and leads to a selectivity greater than 1 at equilibrium. The respective K^+^ selectivity curves indicate that this extended process model successfully captures excluded ion volume effects. The varying adjustable constant *C* (see Equation (4)) from 1.15 to 1.35 is within the range of *C* values determined from experimental observations [34].

Figure 1b shows the transient K^+^ selectivity in MCDI. Initially, K^+^ selectivity in MCDI is lower compared to that in CDI since K^+^ and Na^+^ transport through the IEM are similar and the IEM controls transport (see Figure S1c,d). Excluded ion volume effects only appear after some time due to increased adsorption on the electrodes. After electrode saturation is reached (times greater than 250 s, see Figure S1c,d), the K^+^ selectivity decreases, which is caused by the slow ion penetration through the IEM due to the concentration gradient from electrode macropores to the bulk, causing a repulsion of all ions including K^+^ and Na^+^. Since K^+^ is transported faster than Na^+^, K^+^ selectivity is reduced after electrode saturation.

Figure 1c shows that the selectivity toward the divalent ion Ca^2+^ is significantly greater than for the monovalent species and that excluded ion volume effects can approximately increase selectivity by 50% over that estimated by neglecting those affects. Ca^2+^ selectivity is much higher compared to K^+^ selectivity and continues increasing even after the electrode saturation is achieved (times greater than 200 s, see Figure S1e,f). The increase of Ca^2+^ selectivity after electrode saturation is due to the competitive substitution of Na^+^ by Ca^2+^, which was also observed by Zhao et al. [14].

### 3.2. Trade-Offs between Selectivity and Removal Efficiency of Calcium Ions

**Figure 2:**
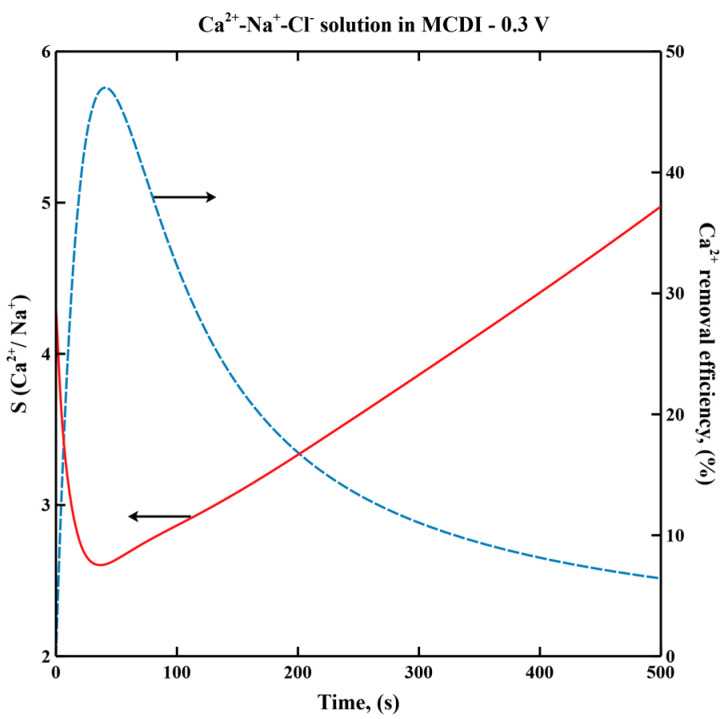
Transient Ca^2+^ selectivity and Ca^2+^ removal efficiency in Ca^2+^-Na^+^-Cl^−^ solution during desalination in MCDI. Feed concentration of each cation is 20 mol/m^3^. Applied voltage is 0.3 V. Ratio of hard sphere diameter to hydraulic diameter, *C* is taken as 1.25.

Figure 2 shows transient Ca^2+^ selectivity and removal efficiency in a Ca^2+^-Na^+^-Cl^−^ solution in MCDI. The trend in Ca^2+^ selectivity is almost opposite to that of Ca^2+^ removal efficiency, indicating a trade-off between selectivity and removal efficiency of Ca^2+^ during desalination. This trade-off was also observed in CC mode MCDI [20]. Although Ca^2+^ selectivity is enhanced by extending desalination operation to near-electrode saturation, overall removal efficiency decreases. To maximize removal efficiency, we propose operating MCDI such that only partial electrode saturation is achieved, i.e., “cut-off” mode [32]. In a multicomponent solution containing Ca^2+^ as the major hardness ions, cut-off mode is defined by cycling MCDI at maximal Ca^2+^ removal efficiency per cycle.

**Figure 3:**
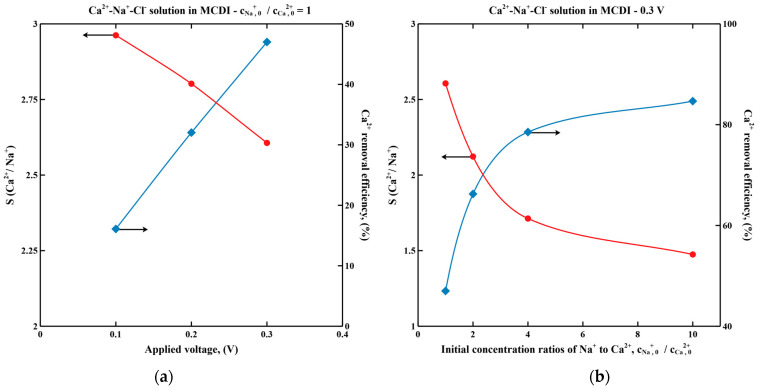
Simulated Ca^2+^ selectivity and Ca^2+^ removal efficiency in Ca^2+^-Na^+^-Cl^−^ solution during desalination in cut-off mode MCDI. (**a**) Feed concentration of each cation is 20 mol/m^3^. Applied voltages are 0.1 V, 0.2 V and 0.3 V, respectively; (**b**) Feed concentration of Na^+^ is 20 mol/m^3^, while feed concentrations of Ca^2+^ are 20 mol/m^3^, 10 mol/m^3^, 5 mol/m^3^ and 2 mol/m^3^, respectively. Applied voltage is 0.3 V. Ratio of hard sphere diameter to hydraulic diameter, *C* is taken as 1.25.

Figure 3 shows the Ca^2+^ selectivity and removal efficiency in Ca^2+^-Na^+^-Cl^−^ solution during desalination in a cut-off mode MCDI under varying applied voltage and initial concentration ratios of cations. By increasing the applied voltage from 0.1 V to 0.3 V, Ca^2+^ selectivity decreases by 20%, while Ca^2+^ removal efficiency increases three-fold due to the increased adsorption capacity at the higher voltage (Figure 3a). Selectivity for Ca^2+^ decreases with increasing feed ratio of Na^+^ to Ca^2+^ (Figure 3b) but Ca^2+^ removal efficiency increases to over 80%.

### 3.3. Case Studies

The feasibility of cut-off CV mode MCDI for softening waters is explored by examining water softening performance and energy consumption for two cases (1) industrial cooling tower blowdown water and (2) domestic tap water.

#### 3.3.1. Industrial Cooling Tower Blowdown Water Softening Scenario

Electrochemical processes [38,39] and pressure-driven membranes [40] have been applied to softening and recycling industrial cooling tower blowdown water to eliminate scaling and reduce the overall water usage. In this case study, the major ion compositions in the cooling tower blowdown water is from Ref. [41] as shown in Table 3. The MCDI is operated in cut-off CV mode with the salt adsorption step operated to achieve maximum salt removal. Water recovery is tuned by adjusting the operating time of the regeneration or desorption step. The operating conditions are shown in Table 2.

The simulated water softening performance including concentration of ionic species in product water, ion removal efficiency, Ca^2+^ selectivity and specific energy consumption (SEC) of quasi-steady state MCDI are shown in Table 3. Here, SEC is based on unit cubic meter of product water and calculated as described in Section S5 of the supporting information. In order to compare with the energy consumed by other water softening techniques, energy consumption per mole of removed Ca^2+^ is given by:

(9) $${\mathrm{SEC}}_{mole}=\frac{\mathrm{SEC}\;}{\Delta c_{C_{a}{}^{2+}}}$$

where Δc_Ca_^2+^ is the concentration reduction of Ca^2+^ in product water during desalination.

Quasi-steady state effluent concentration curves (see Figure S4) are reached within five adsorption/desorption cycles. Under the same water recovery, the removal efficiency of divalent ion is higher than that of monovalent ion with selectivity shown in Table 3. As water recovery increases, overall removal efficiency of each ionic species decreases due to incomplete desorption from the electrodes as a result of shortening of the regeneration/desorption time while Ca^2+^ selectivity increases. The increased product water causes SEC to decrease with increasing water recovery, while SEC*_mole_* varies but can increase at high water recovery since short regeneration/desorption times are used at high water recoveries reducing Ca^2+^ removal. The SEC*_mole_* values in this case study are an order of magnitude less than the reported values from other water softening techniques [39]. Overall, MCDI is energy efficient for partially softening industrial cooling tower blowdown water under moderate water recovery.

**Figure 4:**
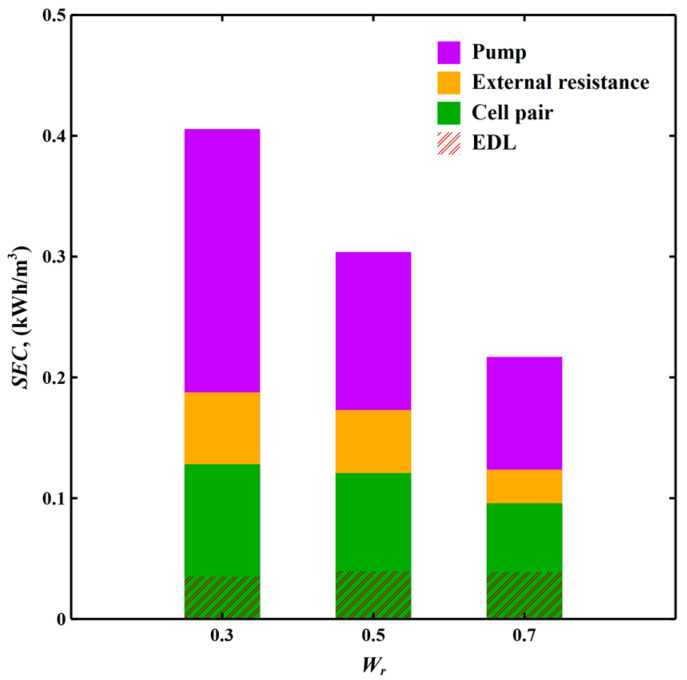
Specific energy consumption (SEC) with the contribution of each component including pump, external resistance and cell pair and the energy stored in electric double layer (EDL) with varying water recovery under quasi-steady state in cut-off constant voltage (CV) mode MCDI for partially softening industrial cooling tower blowdown water. Flow rate is 0.35 L/hr. Applied voltage is 0.4 V.

Figure 4 shows the major components of SEC including energy consumption of pump, external resistance and cell pair and the energy stored in EDL under fractional water recoveries of 0.3, 0.5 and 0.7.

Pump losses are the major energy losses, especially at low water recovery. Since water recovery is tuned by adjusting operating time of regeneration step, lower water recovery indicates longer regeneration and reduced water production, increasing relative pump energy consumption. External resistive losses account for 10–20% of the total energy consumption. The recoverable energy is not significantly affected by varying water recovery, so the recoverable energy as a proportion of the SEC increases with water recovery. In order to reduce SEC, permeability of the porous media spacer should be increased to reduce the pressure drop through the porous spacer-filled channel. Meanwhile, external resistance, especially contact resistance of MCDI elements, should be lowered to bring down the resistive losses.

#### 3.3.2. Domestic Tap Water Softening Scenario

Softening domestic tap water helps address scaling issues and enhances the efficacy of soaps. Considering the relatively low hardness in tap water, slight softening is often sufficient and necessary to avoid corrosion [42]. In this case study, a cut-off CV mode MCDI is used for slightly softening domestic tap water with the major mineral compositions from Ref. [43] shown in Table 4. Anions in tap water are assumed to be Cl^−^. Water recovery is also tuned by adjusting the operating time of regeneration while keeping the same flow rate for both desalination and regeneration steps. The operating conditions are shown in Table 2.

The simulated cell performance, such as concentration of ionic species in product water, ion removal efficiency, Ca^2+^ selectivity, Mg^2+^ selectivity and SEC of quasi-steady state MCDI are shown in Table 4. Since a relatively high water recovery is often preferred for tap water treatment, water recovery is set to 0.5 and 0.7. SEC*_mole_* is also based upon unit mole of Ca^2+^ removed.

Under the same water recovery, Ca^2+^ removal efficiency and selectivity are slightly higher than those of Mg^2+^. This is due to the dual effects of the higher feed concentration and smaller hydrated ion radius of Ca^2+^. Increasing water recovery leads to an increase in the selectivity of both Ca^2+^ and Mg^2+^ and a decrease in SEC and SEC*_mole_*, but reduces the removal efficiencies of all ionic species because of incomplete regeneration of the electrodes. SEC is very low for tap water softening. The SEC values are an order of magnitude less than those reported in a tap water softening study via electrochemical process [42]. Hence, MCDI is energy efficient for slightly softening tap water under moderate water recovery.

**Figure 5:**
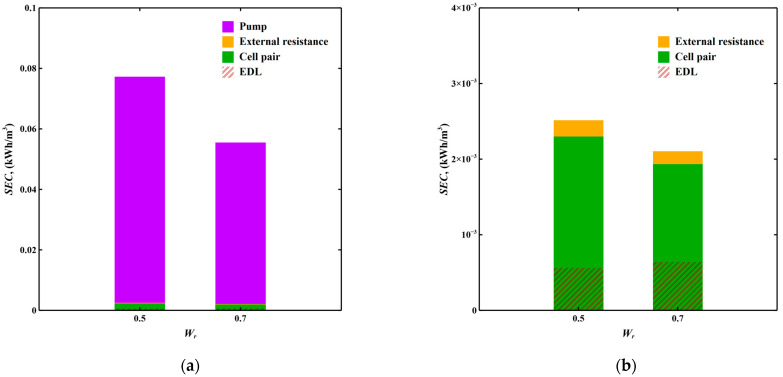
(**a**) SEC with the contribution of each component including pump, external resistance and cell pair and the energy stored in EDL with varying water recovery under quasi-steady state in cut-off CV mode MCDI for slightly softening domestic tap water; (**b**) SEC without pump losses. Applied voltage is 0.08 V.

Figure 5 shows the major components of SEC including energy consumption of pump, external resistance and cell pair and the energy stored in EDL under water recovery of 0.5 and 0.7.

Although increasing water recovery shortens the operating time of regeneration and thus reduces pump losses, pump losses still account for 95% of the energy usage in these simulations although the permeability of the cell spacer can influence that amount. External resistive losses are negligible due to the low external current during desalination, which is attributed to the low applied voltage together with the high resistance of tap water. The recoverable energy increases slightly with increasing water recovery, but is also negligible compared to the huge pump losses. Hence, when softening super low concentration solution such as tap water, reducing pump losses can significantly enhance energy efficiency.

## 4. Conclusions

In this work, our proposed two-dimensional MCDI process model was extended to incorporate excluded ion volume effects, making it possible to distinguish the adsorption behavior of equally charged ions with different hydrated ion radii as well as the selectivity toward divalent ions. Trade-offs between Ca^2+^ selectivity and Ca^2+^ removal efficiency were observed in a Ca^2+^-Na^+^-Cl^−^ solution under either varying applied voltage or varying initial concentration ratios of cations in a cut-off CV mode MCDI. This extended MCDI model was further applied to evaluating water softening performance of a cut-off CV mode MCDI for industrial cooling tower blowdown water and domestic tap water. The SEC of each case was an order of magnitude less than the reported values from other water softening techniques, indicating MCDI to be energy efficient for partially softening industrial waters and slightly softening tap waters under moderate water recovery. Pump losses become dominant for softening super low concentration solutions, such as tap water. Hence, improving the permeability of the porous spacer to reduce the hydraulic pressure drop can reduce the pump energy and save energy. The proposed model can be applied to predicting water softening performance for saline waters with low content of foulants. Pretreatment is required for waters with high content of foulants. The proposed model should be modified to incorporate the effects of Faradaic reactions to predict water softening performance under relatively high applied voltage.

## Figures and Tables

**Table 1:** Common components in brackish waters and their hydrated radii and diffusion coefficients in water.

Components	Hydrated Radii, (nm) [36]	Diffusion Coefficients, (m^2^/s) [37]
Na^+^	0.358	1.33 × 10^−9^
K^+^	0.331	1.96 × 10^−9^
Ca^2+^	0.412	0.79 × 10^−9^
Mg^2+^	0.428	0.71 × 10^−9^
Cl^−^	0.332	2.03 × 10^−9^
NO_3_^−^	0.335	1.90 × 10^−9^
SO_4_^2−^	0.379	1.07 × 10^−9^

**Table 2:** Membrane capacitive deionization (MCDI) device parameters and operating conditions based on a single cell unit.

Parameter	Value	Unit
Cell length	10	[cm]
Cell width	10	[cm]
Electrode thickness	0.15	[mm]
Macropore porosity	0.4 [27]	-
Micropore porosity	0.3 [27]	-
Micropore capacitance	1.5 [27]	[GF/m^3^]
IEM thickness	0.15	[mm]
IEM water content volume fraction	0.4	[L(water)/L(swollen IEM)]
IEM fixed charge density	1000	[mol/m^3^]
Spacer-filled channel thickness	0.3	[mm]
Spacer porosity	0.71	-
Spacer permeability	1.23 × 10^−12^	[m^2^]
External resistance	0.6	[Ω]
C = *d_i_/d_h,i_*	1.15–1.35, 1.25 * ^#^ [34]	-
Flow rate	0.3, 0.35 *, 0.2 ^#^	[L/h]
Applied voltage	0.1–0.3, 0.4 *, 0.08 ^#^	[V]

* Parameters used in industrial cooling tower blowdown water softening example, Section 3.3.1. ^#^ Parameters used in residential tap water softening example, Section 3.3.2.

**Table 3:** Water softening performance and energy behaviors of quasi-steady state MCDI for partially softening industrial cooling tower blowdown water.

Parameter		Value		
Concentration of ionic species in the feed water, (mM) [41]	Na^+^	24.35		
	Ca^2+^	7.48		
	Cl^−^	14.95		
	NO_3_^−^	0.98		
	SO_4_^2−^	11.69		
		Water recovery		
		0.3	0.5	0.7
Concentration of ionic species in the product water, (mM)	Na^+^	13.21	17.44	22.64
	Ca^2+^	1.28	2.50	5.19
	Cl^−^	9.23	11.53	14.19
	NO_3_^−^	0.62	0.76	0.93
	SO_4_^2−^	2.96	5.07	8.95
Ion removal efficiency, (%)	Na^+^	45.76	28.37	7.03
	Ca^2+^	82.88	66.64	30.62
	Cl^−^	38.25	22.89	5.09
	NO_3_^−^	37.21	22.37	5.22
	SO_4_^2−^	74.68	56.61	23.43
Ca^2+^ selectivity		1.81	2.35	4.36
SEC, (kWh/m^3^)		0.406	0.304	0.217
SEC*_mole_*, (kWh/mole)		0.065	0.061	0.095

**Table 4:** Water softening performance and energy behaviors of quasi-steady state MCDI for slightly softening domestic tap water.

Parameter		Value	
Concentration of ionic species in the feed water, (mM) [43]	Na^+^	1.65	
	Ca^2+^	0.75	
	K^+^	0.13	
	Mg^2+^	0.38	
	Cl^−^	4.04	
		Water recovery	
		0.5	0.7
Concentration of ionic species in the product water, (mM)	Na^+^	1.44	1.58
	Ca^2+^	0.53	0.66
	K^+^	0.11	0.13
	Mg^2+^	0.27	0.34
	Cl^−^	3.16	3.71
Ion removal efficiency, (%)	Na^+^	12.94	4.38
	Ca^2+^	28.90	11.43
	K^+^	12.80	1.63
	Mg^2+^	28.58	10.78
	Cl^−^	21.80	8.12
Ca^2+^ selectivity		2.23	2.61
Mg^2+^ selectivity		2.21	2.46
SEC, (kWh/m^3^)		0.077	0.055
SEC*_mole_*, (kWh/mole)		0.356	0.256

## Data Availability

The data presented in this study are available on request from the corresponding author.

## Supplementary Materials

The following are available online at https://www.mdpi.com/2077-0375/11/4/231/s1.
