# Supplementary material for: Theoretical Analysis of Constant Voltage Mode Membrane Capacitive Deionization for Water Softening
Source: Membranes (Basel). 2021 Mar 24;11(4):231. doi: 10.3390/membranes11040231 (PMC8064386; doi:10.3390/membranes11040231)
Supplement: Supplementary file 1 [file membranes-11-00231-s001.pdf]

membranes

Supplementary Materials: Theoretical Analysis of Constant Voltage Mode Membrane Capacitive Deionization for Water Softening

Xin Zhang[a], and Danny Reible[a,b,*]

[a] Department of Chemical Engineering, Texas Tech University, Lubbock, TX 79409-3121, USA
[b] Department of Civil, Environmental, and Construction Engineering, Texas Tech University, Lubbock, TX 79409-1023, USA
* Correspondence: Corresponding author. Tel.: +1 806.834.8050. Email address: *danny.reible@ttu.edu*

SUMMARY

Number of pages: 9
Number of Figures: 5

S1. Transient effluent concentration curves of cations in K^+-Na^+-Cl^- solution with and without considering excluded ion volume effects

Transient effluent concentration curves of cations with and without excluded ion volume effects during desalination in (M)CDI are displayed in Figure S1. In K^+-Na^+-Cl^- solution, CDI approaches equilibrium within 400 s, which is almost twice as long as MCDI. However, Ca^{2+}-Na^+-Cl^- solution takes longer time to reach equilibrium due to the competitive substitution of Na^+ by Ca^{2+} even after electrode saturation is achieved. Transient effluent concentration curves with and without considering excluded ion volume effects have negligible differences in both CDI and MCDI. The transport and adsorption rate of K^+ is a bit faster compared to that of Na^+ in CDI but is almost the same as that of Na^+ in MCDI. This is due to the equally enhanced transport rate of both cations through IEM due to the same valence of K^+ and Na^+.

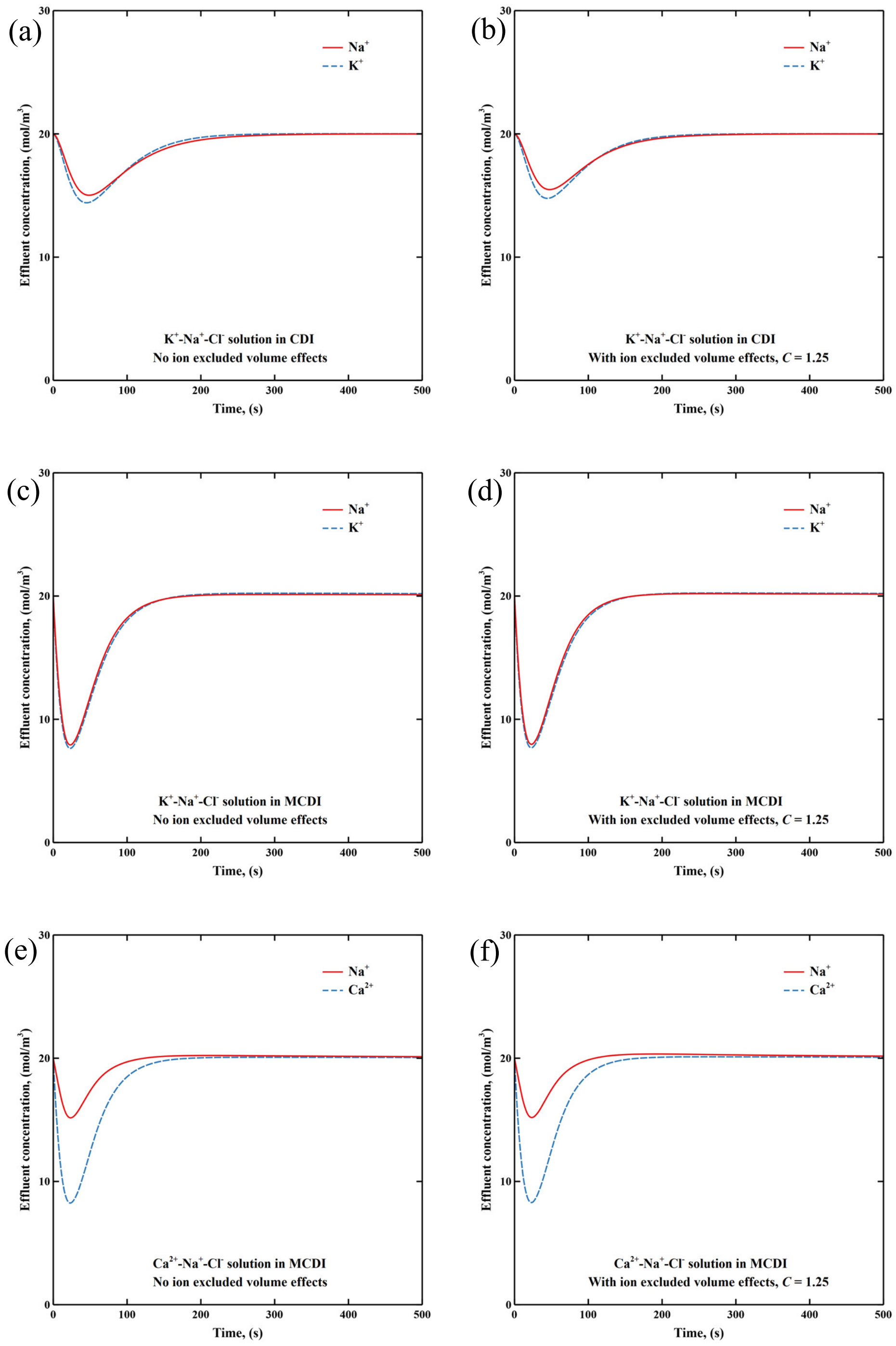

Figure S1. Transient effluent concentration curves of cations in K^+-Na^+-Cl^- solution in CDI (**a**) without ion excluded volume effects, (**b**) with ion excluded volume effects (C = 1.25); Transient effluent concentration curves of cations in K^+-Na^+-Cl^- solution in MCDI (**c**) without ion excluded volume effects, (**d**) with ion excluded volume effects (C = 1.25); Transient effluent concentration curves of cations in Ca^{2+}-Na^+-Cl^- solution in MCDI (**e**) without ion excluded volume effects, (**f**) with ion excluded volume effects (C = 1.25); Applied voltage is 0.3 V. Feed concentration of each cation is 20 mol/m^3.

S2. Transient effluent concentration curves of cations in Ca^{2+}-Na^{+}-Cl^{-} solution under varying applied voltage

The simulated transient effluent concentration curves of cations in Ca^{2+}-Na^{+}-Cl^{-} solution under varying applied voltage of 0.1 V, 0.2 V and 0.3 V during desalination in MCDI are displayed in Figure S2. The adsorption of both cations increase with applied voltage due to the increased adsorption capacity of the electrode. The adsorption amount of Ca^{2+} is much higher than Na^{+}. The effluent concentration of Na^{+} goes slightly beyond its initial concentration when electrode saturation is approached, indicating the occurrence of a competitive substitution of Na^{+} by Ca^{2+}. This phenomena is more significant under a higher applied voltage.

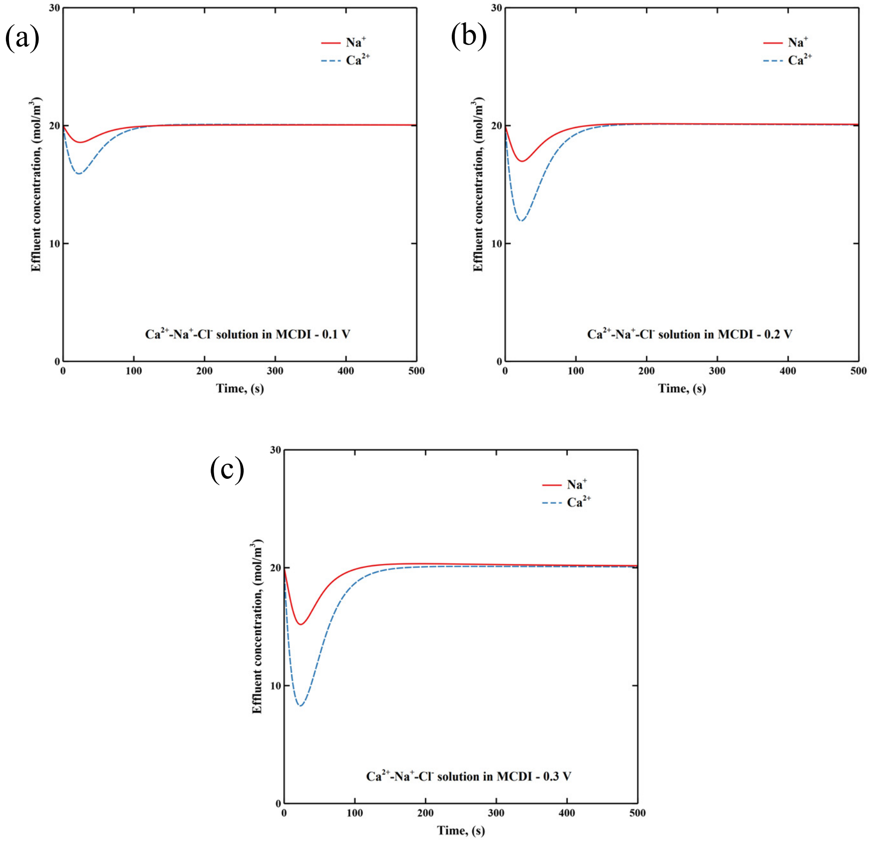

Figure S2. Simulated transient effluent concentration curves of cations in Ca^{2+}-Na^{+}-Cl^{-} solution under varying applied voltage of (**a**) 0.1 V, (**b**) 0.2 V, and (**c**) 0.3 V during desalination in MCDI. The adjustable variable C is set to 1.25. Feed concentration of each cation ion is 20 mol/m^3.

S3. Transient effluent concentration curves of cations in Ca^{2+}-Na^{+}-Cl^{-} solution under varying initial concentration ratios of cations

The simulated transient effluent concentration curves of cations in Ca^{2+}-Na^{+}-Cl^{-} solution under varying initial concentration ratios of cations during desalination in MCDI are displayed in Figure S3. The initial concentration ratio of cations is tuned by adjusting the feed concentration of Ca^{2+} from 20

mol/m³ to 2 mol/m³ and fixing the feed concentration of Na^+ at 20 mol/m³ at the same time. As the initial concentration ratio of Na^+ to Ca^{2+} increases, the adsorption amount of Na^+ increases, while the adsorption amount of Ca^{2+} decreases. Meanwhile, the competitive substitution of Na^+ by Ca^{2+} slows the return of Na^+ to its initial concentration.

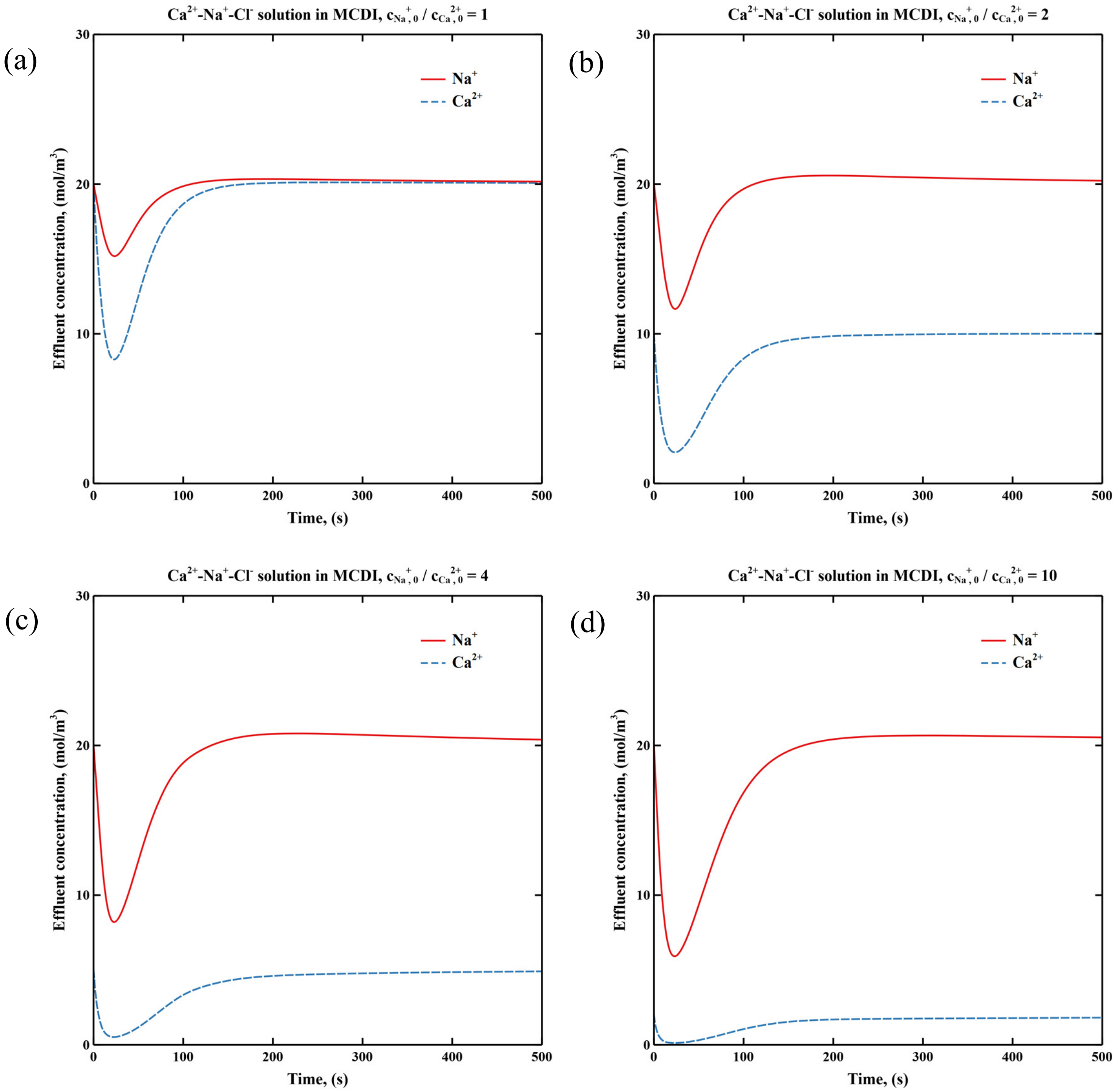

Figure S3. Simulated transient effluent concentration curves of cations under varying initial concentration ratios of cations at (**a**) $c_{Na^+,0}/c_{Ca^{2+},0} = 1$, (**b**) $c_{Na^+,0}/c_{Ca^{2+},0} = 2$, (**c**) $c_{Na^+,0}/c_{Ca^{2+},0} = 4$ and (**d**) $c_{Na^+,0}/c_{Ca^{2+},0} = 10$ in Ca^{2+}-Na^+-Cl^- solution during desalination in MCDI. The adjustable variable C is set to 1.25. Applied voltage is 0.3 V.

S4. Transient effluent concentration curves of cations and anions in MCDI when softening industrial cooling tower blowdown water and domestic tap water

The simulated transient effluent concentration curves of cations and anions in five consecutive cycles containing Na^+, Ca^{2+}, Cl^-, NO_3^- and SO_4^{2-} under

varying water recovery from 0.3 to 0.7 in cut-off CV mode MCDI for softening industrial cooling tower blowdown water are displayed in Figure S4. A full cycle includes a desalination step followed by a regeneration step. In this study, the regeneration of MCDI is the result of short-circuiting the cell. MCDI reaches quasi-steady state within five consecutive cycles. Water recovery is tuned by adjusting the operating time of regeneration step and fixing the flow rate for both desalination and regeneration steps. As water recovery increases, the desalination capacity of MCDI decreases due to the incomplete regeneration of the electrodes, causing decreased adsorption of all ions. The effects of fraction water recovery on ion adsorption are more significant for monovalent ions compared to divalent ions.

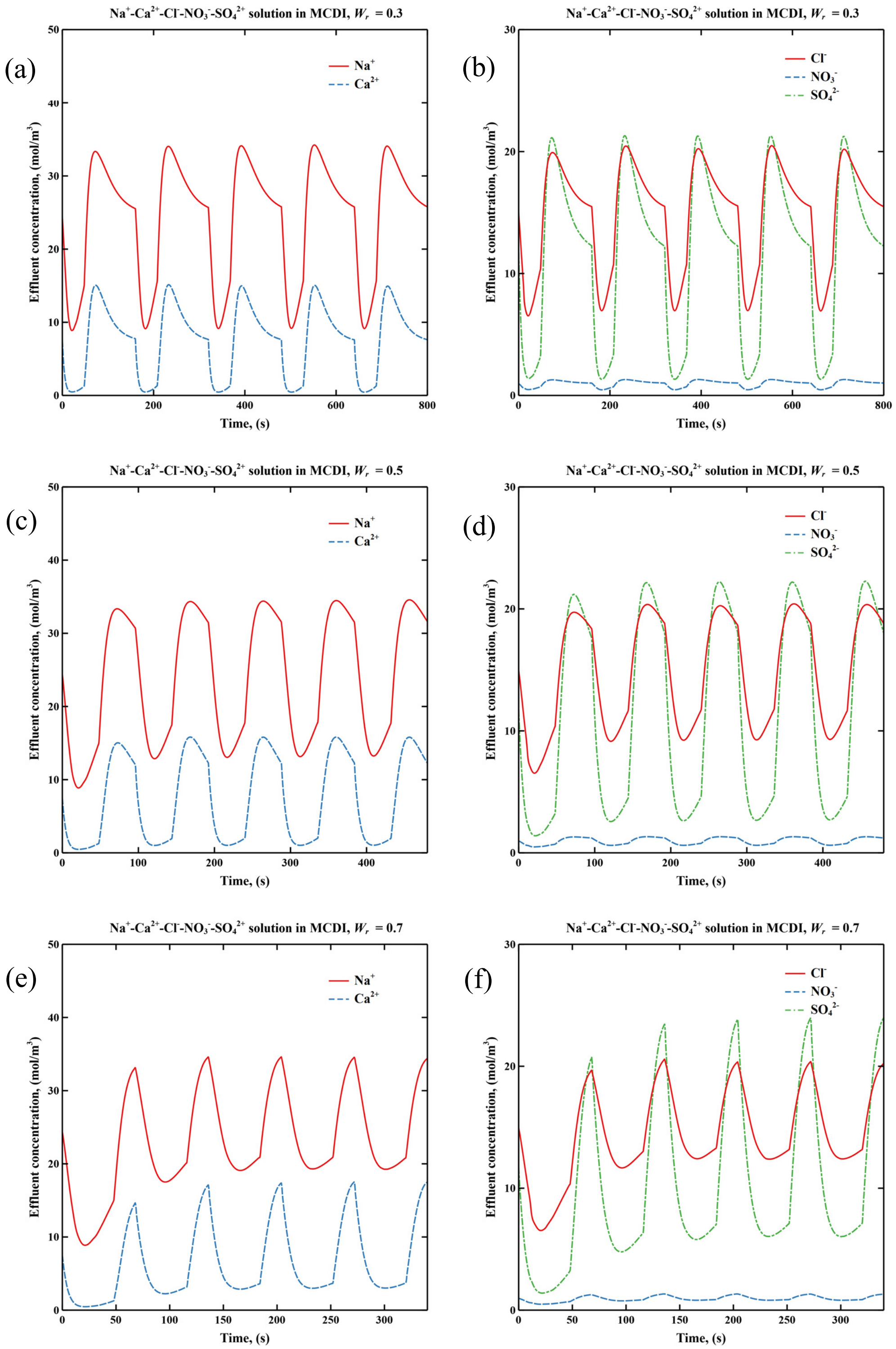

Figure S4. Simulated transient effluent concentration curves of cations under water recovery of (**a**) 0.3, (**c**) 0.5, and (**e**) 0.7, respectively, and anions under water recovery of (**b**) 0.3, (**d**) 0.5, and (**f**) 0.7, respectively, in five consecutive cycles in cut-off CV mode MCDI when softening industrial cooling tower blowdown water. The adjustable variable *C* is set to 1.25. Applied voltage is 0.4 V.

The simulated transient effluent concentration curves of cations and anions in five consecutive cycles containing Na^{+}, Ca^{2+}, K^{+}, Mg^{2+} and Cl^{-} under varying water recovery from 0.5 to 0.7 in cut-off CV mode MCDI for softening domestic tap water are displayed in Figure S5. MCDI reaches quasi-steady state within five consecutive cycles. The concentrations of ionic species in domestic tap water are low compared to those in industrial cooling tower blowdown water. So a small applied voltage can meet the requirement of partial desalination. In this case study, the increase of water recovery also reduces ion adsorption due to incomplete regeneration of the electrodes due to the shortened regeneration time.

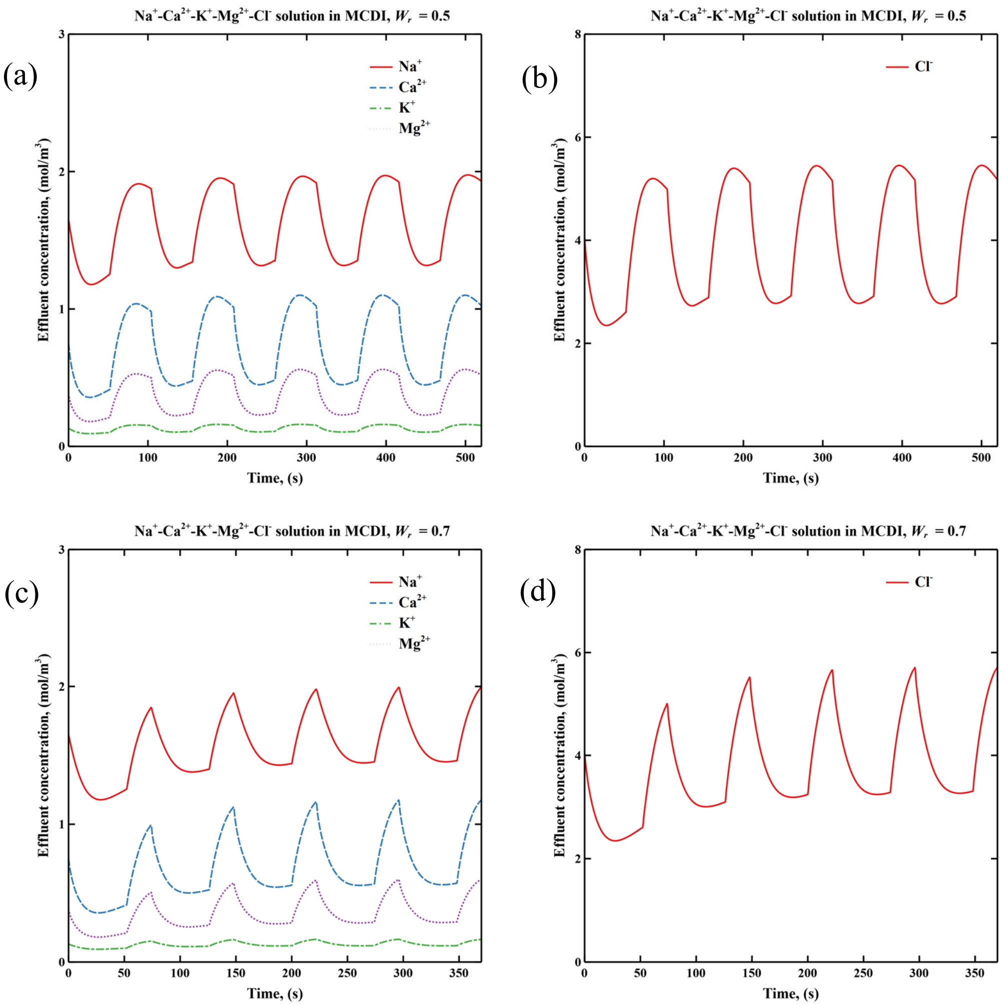

Figure S5. Simulated transient effluent concentration curves of cations under water recovery of (**a**) 0.5 and (**c**) 0.7, and anions under water recovery of (**b**) 0.5 and (**d**) 0.7, in five consecutive cycles in cut-off CV mode MCDI for softening domestic tap water. The adjustable variable *C* is set to 1.25. Applied voltage is 0.08 V.

S5. Energy consumption components of MCDI

Energy consumption in MCDI include energy consumed for charging the electrodes and pumping the flowing solution. In this study, the components of energy consumption include pump losses, external resistive losses and cell pair energy consumption, and are expressed as a specific energy consumption (SEC) form by dividing the volume of product water.

Pump losses represent the pump energy used for pumping feed water through the porous spacer-filled channel in both desalination and regeneration steps. Energy consumption of pump is given by:

$$E_{pump} = \frac{\sum_{j=d,r} \Delta P_j Q_j t_j}{Q_d t_d} \quad (1)$$

Copyright: © 2021 by the authors. Licensee MDPI, Basel, Switzerland. This article is an open access article distributed under the terms and conditions of the Creative Commons Attribution (CC BY) license (http://creativecommons.org/licenses/by/4.0/).

where ΔP_j, Q_j, and t_j are the pressure drop through the channel, flow rate, and operating time of operating step, *j*, including desalination step, *d*, and regeneration step, *r*, respectively.

Pressure drop through the porous channel is given by:

$$\Delta P_i = \frac{\mu Q_i L}{k A_c} \quad (2)$$

Where μ is the viscosity of feed water, *L* is the cell length, *k* is the permeability of the porous spacer, and A_c is the cross-sectional area of the channel, width times thickness ($W \times L_{ch}$).

External resistive losses account for energy losses on contact resistance between current collector and electrode, wire resistance and current collector resistance, and can be expressed by:

$$E_{ext} = \frac{\int_0^{t_d} I_{ext}^2 R_{ext} dt}{Q_d t_d} \quad (3)$$

(4)

Energy consumed in cell pairs is used for ion transport and adsorption in the cell, and is given by:

$$E_{cell} = E_{eps} - E_{ext} = \frac{\int_0^{t_d} V_{cell} I_{ext} dt}{Q_d t_d} - E_{ext} \quad (5)$$

where V_{cell} is the applied voltage including the potential drop on the external resistance and the cell pair.

The total SEC is the sum of the three components mentioned above:

$$SEC = E_{pump} + E_{ext} + E_{cell} \quad (6)$$

Along with ion adsorption, part of the input energy is temporarily stored in EDL. This energy is the maximum recoverable energy and can be partially harvested during regeneration. This energy storage amount is given by [1]:

$$E_{EDL} = \frac{\int_0^{t_d} I_{ext}(\varphi_{st} + \varphi_D)dt}{Q_d t_d} \quad (7)$$

where φ_{st} is the Stern layer potential drop, and φ_D is the Donnan potential.

References

1. Salamat, Y. and C.H. Hidrovo, *A parametric study of multiscale transport phenomena and performance characteristics of capacitive deionization systems.* Desalination, 2018. **438**: p. 24-36.
